# Are Linear Cephalometric Measurements Interpreted Equally Across Birth Cohorts? Cross-Sectional Cephalometric Study

**DOI:** 10.3390/dj14040194

**Published:** 2026-03-25

**Authors:** Luis Pablo Cruz-Hervert, Luis Cruz-Chávez, Gerardo Martínez-Suárez, Carla Monserrat Ramírez-Martínez, Alvaro Édgar González-Aragón Pineda, Socorro Aída Borges-Yánez, Beatriz Raquel Yáñez-Ocampo, Jaqueline Adelina Rodríguez-Chávez, Álvaro García-Pérez, Janet Real-Ramírez, Sergio Sánchez-García, María-Eugenia Jiménez-Corona, Luis Fernando Jacinto-Alemán

**Affiliations:** 1 División de Estudios de Posgrado e Investigación, Facultad de Odontología, Universidad Nacional Autónoma de México, Ciudad de México 04510, Mexico; dr.luis.cruz@gmail.com (L.C.-C.); c.ramirez@fo.odonto.unam.mx (C.M.R.-M.); aborges@unam.mx (S.A.B.-Y.); raquel.yaez@gmail.com (B.R.Y.-O.); mejimenez777@gmail.com (M.-E.J.-C.); 2 Departamento de Epidemiología, Instituto Nacional de Cardiología Ignacio Chávez, Ciudad de México 14080, Mexico; 3 Facultad de Estudios Superiores (FES) Iztacala, Universidad Nacional Autónoma de México, Tlalnepantla de Baz 54090, Mexico; drsgema@yahoo.com.mx (G.M.-S.); alvaroedgar@unam.mx (A.É.G.-A.P.); alvaro.garcia@unam.mx (Á.G.-P.); 4 Departamento de Clínicas Odontológicas Integrales, Centro Universitario de Ciencias de la Salud, Universidad de Guadalajara, Guadalajara 44340, Mexico; jacqueline.rchavez@academicos.udg.mx; 5 Departamento de Innovación Educativa en Salud, Instituto Nacional de Enfermedades Respiratorias Ismael Cosío Villegas, Ciudad de México 14080, Mexico; janet.real@iner.gob.mx; 6 Unidad de Investigación Epidemiológica y en Servicios de Salud, Área Envejecimiento, Centro Médico Siglo XXI, Instituto Mexicano del Seguro Social, Ciudad de México 06720, Mexico; sergio.sanchezga@imss.gob.mx

**Keywords:** linear cephalometric measurements, cephalometric norms, craniofacial dimensions, craniofacial structures, birth cohort, birth cohort association

## Abstract

**Background/Objectives**: This study evaluated whether linear cephalometric measurements show systematic differences in their central values across birth cohort groups in adults from a clinical population and analyzed the implications of these differences for clinical interpretation when norms and clinical deviations are used as a reference framework. **Methods**: A cross-sectional observational analytical study was conducted based on 604 lateral cephalometric radiographs of adult patients. Eleven linear cephalometric measurements were obtained and compared across predefined birth cohort groups (<1980, 1980–1989, and 1990–1999) using robust estimators of central tendency through median regression models adjusted for sex, age group, and sagittal skeletal classification. **Results**: Several linear cephalometric measurements revealed different central values between the birth cohorts, even after adjusting for relevant covariates. Cranial length, anterior cranial base length, posterior facial height, and posterior cranial base length had lower adjusted median values in the 1990–1999 cohort than in the <1980 cohort. The effective maxillary length and maxillary length also differed between cohorts. Mandibular measurements, including mandibular length, corpus length, and ramus height, showed the largest adjusted median contrasts between cohorts. These cohort-associated differences were not uniform across all measurements. **Conclusions**: Routinely used linear cephalometric measurements present different central values across adult birth cohort groups under comparable clinical conditions. The relative position of a cephalometric value within its reference distribution may vary by birth cohort. This suggests that using fixed reference means and standard deviations could lead to systematic misestimation in adults from various birth cohorts. Cohort-aware interpretation is valuable in routine cephalometric assessments.

## 1. Introduction

Linear cephalometric measurements are routinely used in orthodontic diagnosis to determine whether craniofacial dimensions fall within the expected clinical limits. This interpretation depends on a comparison with reference means and standard deviations derived from historical samples, which are commonly assumed to represent stable diagnostic baselines [1,2,3]. However, patients treated today arise from different biological and population contexts, raising the possibility that identical measurements may not occupy the same relative position within the reference framework across birth cohort groups [4,5].

Because cephalometric interpretation is inherently relative, a measurement acquires meaning through its position within a reference distribution rather than its absolute magnitude alone. If contemporary patients differ from the populations used to construct the norms, the same value may be classified differently without implying a true structural discrepancy in the population. Such differences represent potential interpretive inconsistencies rather than biological changes and may influence clinical categorization in heterogeneous patient populations [4,5,6,7,8,9].

Previous research has described intergenerational variation in craniofacial dimensions, often referred to as secular variation, although the findings are heterogeneous and not consistently directional [6,7,8,9]. Some dimensions appear higher, others lower, and some are stable depending on the anatomical region and measurement type. These observations suggest that variation is selective across measurements rather than uniform across the craniofacial complex [6,8,9,10]. From a diagnostic perspective, this raises the question of whether each cephalometric variable preserves equivalent interpretive meaning across birth cohort groups.

In clinical cephalometry, linear measurements quantify specific anatomical dimensions, whereas angular measurements describe the positional relationships between structures. Accordingly, each linear variable functions as an independent diagnostic construct rather than as part of a single global craniofacial pattern. Therefore, evaluating multiple measurements examines the stability of their interpretive framework rather than repeated assessment of the same phenomenon [3,9,10].

To evaluate temporal associations while avoiding confounding by growth processes, comparisons can be performed across birth cohort groups, defined as individuals born within a specific period who share similar early-life exposures and population contexts [11]. Because it would be unethical to diagnose individuals with growth disturbances or malocclusions and deliberately leave them untreated to obtain subsequent observations, true untreated longitudinal records are rarely available in orthodontic clinical data. Therefore, birth cohort group analysis provides a practical strategy for assessing intergenerational associations while maintaining the adult developmental stage constant [12,13].

The aim of this study was to evaluate whether commonly used linear cephalometric measurements show different distributions across adult birth cohort groups and to examine the implications of these associations for clinical interpretation based on conventional reference norms. When examined using multivariable models, several contrasts persisted after adjusting for sex, age group, and sagittal skeletal classification.

## 2. Materials and Methods

### 2.1. Study Design

This was a cross-sectional observational analytical study based on retrospective orthodontic diagnostic records. This design allowed for the comparison of linear cephalometric measurements across predefined birth cohort groups in adults. Given the cross-sectional nature of the data, this study did not evaluate longitudinal changes, growth patterns, or within-individual trajectories.

### 2.2. Study Population

The study population comprised adult patients who underwent routine lateral cephalometric radiography as part of the comprehensive orthodontic diagnostic records at a single clinical center. Only individuals aged ≥18 years were included to minimize the influence of active craniofacial growth, as residual changes after this stage are considered minimal. All records were derived from standard clinical care and retrospectively retrieved from an orthodontic clinical database containing approximately 2500 available records. Records were selected based on predefined eligibility criteria that were uniformly applied across the database.

### 2.3. Sample Size Considerations

From the available database, 604 lateral cephalograms met the eligibility criteria and were included in the final analysis. An a priori sample size estimation was performed for multivariable regression analyses, assuming an alpha level of 0.05, statistical power of 0.90, and up to 15 potential predictors, following general recommendations for multivariable modeling. Under these assumptions, the selected sample size was considered sufficient to detect clinically relevant cohort-related differences in linear cephalometric measurements with adequate precision.

### 2.4. Inclusion and Exclusion Criteria

Records were eligible for inclusion if the patients were ≥18 years of age at the time of radiographic acquisition and if a complete set of predefined linear cephalometric measurements could be obtained. Records were excluded if the radiographs showed craniofacial syndromes, congenital anomalies, a history of craniofacial or orthognathic surgery, or traumatic alterations affecting craniofacial structures. Records with incomplete demographic data or insufficient radiographic quality that precluded reliable landmark identification were also excluded. Each record was included once, and no repeated records were analyzed per individual.

### 2.5. Birth Cohort Groups

Birth cohort groups were defined a priori according to the year of birth and categorized as <1980, 1980–1989, and 1990–1999. These categories were established before the analysis, reflected meaningful temporal groupings within the available dataset, and were consistently used throughout the study. For analytical purposes, the <1980 cohort was used as the reference category in the multivariable regression models.

### 2.6. Cephalometric Variables

**Figure 1:**
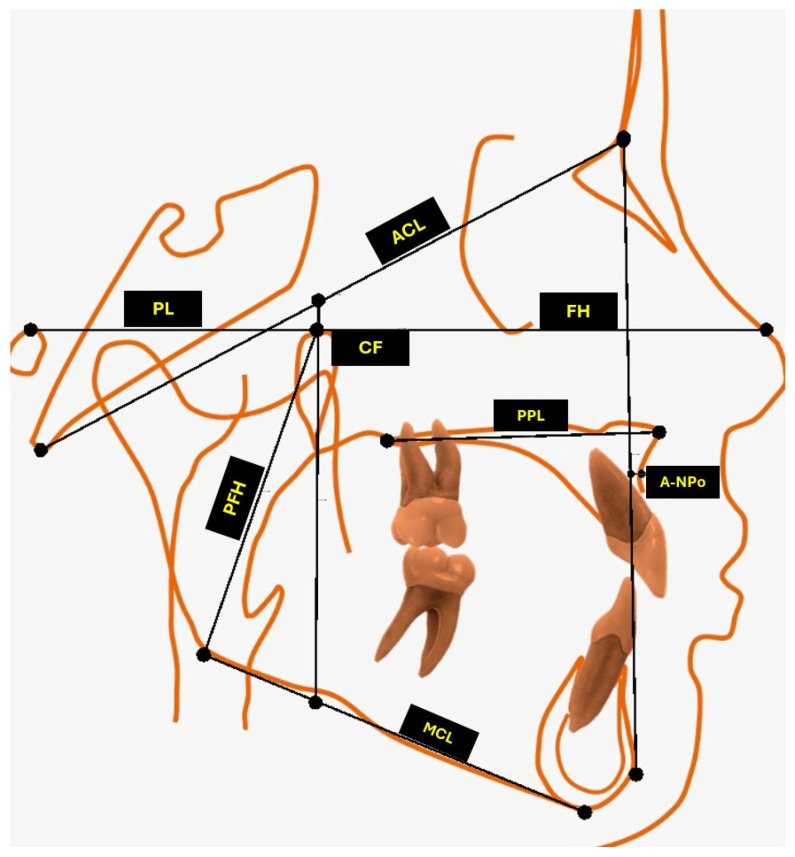
Schematic description of linear cephalometric measurements. Footnote: PL, Porion Location; ACL, Anterior Cranial Length; FH, SN, Anterior Cranial Base; CF, Cranial Floor; PPL, Palatal Plane (PNS–ANS); PFH, Posterior Facial Height (Go–CF); MCL, Go–Gn Mandibular Corpus Length; A–NPo, Convexity; A–NPo; S–Ar, Posterior Cranial Base; Co–A, Maxillary Length; Co–Gn, Mandibular Length; Ar–Go, Ramus Height.

Eleven linear cephalometric measurements were obtained from each record for the analyses. The analyzed variables were cranial length, anterior cranial base (S–N), posterior cranial base (S–Ar), posterior facial height (Go–CF), porion location, maxillary length (Co–A), PNS–ANS, convexity (A–NPo), mandibular length (Co–Gn), corpus length (Go–Gn), and ramus height (Ar–Go). Each measurement represented a distinct cranial, maxillary, mandibular, or vertical dimension and was expressed in millimeters. Landmark identification and measurement definitions followed established cephalometric standards and are summarized in Table 1 and Figure 1.

### 2.7. Covariates and Skeletal Classification

Sex was recorded as female or male, as appropriate. Age at the time of radiographic acquisition was categorized into four groups: 18–29, 30–39, 40–49, and ≥50 years. Skeletal classification was determined using the ANB angle and categorized according to conventional orthodontic thresholds as Class I (ANB 0–4°), Class II (ANB > 4°), or Class III (ANB < 0°). The ANB angle was used as a conventional clinical categorization tool rather than as a measure of the integrated craniofacial morphology. These variables were included as covariates in the analytical models to account for clinically relevant variations in linear cephalometric measurements.

### 2.8. Cephalometric Procedures

All lateral cephalometric radiographs were obtained under standardized clinical conditions, following routine orthodontic diagnostic protocols. All radiographs were obtained using the same cephalometric radiographic equipment. Cephalometric tracings and landmark identification were performed digitally using Dolphin Imaging Software version 11.0 (Imaging and Management Solutions, Chatsworth, CA, USA) ) by a single calibrated examiner. Linear measurements were recorded directly from the digital tracings in millimeters using consistent measurement definitions across the full dataset.

### 2.9. Measurement Reliability

Measurement reliability was assessed using a random subsample of 30 lateral cephalometric radiographs. The radiographs were re-evaluated after an interval of approximately 30 days by the same examiner and independently by a second orthodontist. Intra- and inter-examiner agreement were quantified using intraclass correlation coefficients (ICCs). All measurements demonstrated excellent reproducibility, with ICC values exceeding 0.88, thus supporting their use in subsequent analyses.

### 2.10. Statistical Analysis

All statistical analyses were conducted using Stata software (version 15.1; StataCorp, College Station, TX, USA). Continuous variables were summarized using medians and interquartile ranges, and categorical variables were summarized using frequencies and percentages. The analytical workflow followed a predefined sequence: (1) descriptive characterization of the study population and cephalometric measurements; (2) analytical comparison of cohort-related differences via robust estimators of central tendency using median (50th percentile) regression models; (3) evaluation of distributional consistency using quantile regression models at the 25th, 50th, and 75th percentiles; and (4) graphical descriptive assessment of cohort-specific measurement distributions relative to established clinical norms and their corresponding standard deviation thresholds. This sequence was predefined to separate descriptive characterizations from analytical contrasts and to avoid post hoc analytical decisions.

Multivariable quantile regression models were fitted for each linear cephalometric measurement using the <1980 cohort as the reference category, adjusting for sex, age group, and sagittal skeletal classification. All quantile regression models were estimated using bootstrap procedures with 500 replications to obtain robust standard errors and confidence intervals. Statistical significance was set at *p* < 0.05 (two-sided). Each cephalometric measurement was analyzed as an independent variable in the study. No correction for multiple comparisons was applied because all outcomes were pre-specified and represented distinct clinical constructs.

### 2.11. Ethical Considerations

The study protocol was reviewed and approved by the Ethics and Research Committee of the Faculty of Dentistry, National Autonomous University of Mexico (approval no. CIE02/10/06/2016/04). All data were anonymized prior to analysis.

## 3. Results

The primary finding of this study was that several linear cephalometric measurements showed different central values between birth cohort groups in adults, even after adjusting for sex, age group, and sagittal skeletal classification group. These differences were observed as cross-sectional contrasts between cohorts, expressed as higher, lower, or similar values in the distribution of specific measurements of the variables. They do not represent longitudinal changes, growth, or variations within individuals. Accordingly, all results were interpreted at the level of cephalometric measurements as clinical units of analysis rather than as trajectories of individual participants.

### 3.1. Characteristics of the Study Population

A total of 604 adult cephalometric records were analyzed, with each record contributing to a complete set of linear cephalometric measurements. The distributions of age, sex, birth cohort, and sagittal skeletal classification (ANB categories) are presented in Table 1. The sample was predominantly composed of individuals aged 18–29 years (61.8%), with expected differences in age distribution across birth cohorts. In contrast, the distributions of sex and skeletal class were comparable across cohorts, with females representing 68.4% of the total sample and Class II malocclusion accounting for 81.5% of all records. These similarities support the comparison of the measurement distributions across birth cohorts within a consistent clinical and diagnostic context.

### 3.2. Descriptive Linear Cephalometric Measurements by Birth Cohort Group

The descriptive statistics for the 11 prespecified linear cephalometric measurements by birth cohort group are presented in Table 2 as medians and interquartile ranges. At the descriptive level, several measurements revealed differences in the central tendency across cohorts, whereas others displayed substantial overlap in the interquartile ranges.

Overall, the <1980 and 1980–1989 cohorts presented similar median values for most of the measurements. In contrast, the 1990–1999 cohort presented lower median values for several cranial base, maxilla, and mandible dimensions. For example, the median cranial length decreased from 56.2 mm in the <1980 cohort to 52.4 mm in the 1990–1999 cohort, whereas the median anterior cranial base length (S–N) differed by more than 3 mm between these cohorts. Similar distributional shifts were observed for the effective maxillary length (Co–A), mandibular length (Co–Gn), and corpus length (Go–Gn) measurements.

However, not all measurements followed this trend. Convexity (A–NPo) showed overlapping medians and interquartile ranges across cohorts, with a global comparison that did not reach statistical significance (*p* = 0.055). Ramus height also showed overlapping distributions between cohorts, despite the modest differences in median values. These findings indicate that cohort-associated differences are not uniform across all cephalometric constructs, reinforcing the need to interpret each pre-specified measurement.

### 3.3. Associations Between Birth Cohort Groups and Linear Cephalometric Measurements

To quantify cohort-associated differences while accounting for relevant clinical covariates, multivariate quantile regression models were fitted at the 50th percentile for each measurement. The adjusted results are shown in Table 3. In these models, the coefficients represent the differences in the adjusted medians between birth cohorts, with the <1980 cohort used as the reference group. All estimates are reported in millimeters with 95% confidence intervals and describe cross-sectional contrasts, rather than longitudinal changes.

After adjustment, several cranial domain measurements presented lower adjusted median values in the 1990–1999 cohort than in the <1980 cohort. The cranial length differed by −5.2 mm (95% CI −6.8 to −3.7), and the anterior cranial base length (S–N) differed by −5.6 mm (95% CI −7.2 to −4.0). Posterior facial height (Go–CF) and posterior cranial base length (S–Ar) also presented lower adjusted medians in the 1990–1999 cohort, with differences of −4.6 mm and −2.3 mm, respectively. In contrast, porion location had a higher adjusted median value in the 1990–1999 cohort, differing by +4.8 mm (95% CI: 3.2 to 6.4). Taken together, these results indicate that cranial reference measurements differ in direction and magnitude across cohorts rather than shifting uniformly across them.

In the maxillary domain, the effective maxillary length (Co–A) had a lower adjusted median in the 1990–1999 cohort by −7.7 mm (95% CI −10.2 to −5.2), whereas the maxillary length (PNS–ANS) differed by −3.3 mm (95% CI −5.7 to −0.9). In contrast, convexity (A–NPo) did not show evidence of a cohort-associated difference at the adjusted median level, with an estimated difference of −0.3 mm (95% CI −1.5;0.9). This result indicates that some routinely used cephalometric constructs remain comparable across cohorts, even when other linear dimensions differ between groups.

Mandibular measurements showed the largest adjusted median contrast between the cohorts. Compared with the <1980 cohort, the 1990–1999 cohort presented lower adjusted medians for mandibular length (Co–Gn), by −11.8 mm (95% CI −15.2 to −8.4); corpus length (Go–Gn), by −5.4 mm (95% CI −8.4 to −2.4); and ramus height (Ar–Go), by −4.5 mm (95% CI −6.6 to −2.4). These estimates indicate that typical mandibular measurement values differ substantially between cohorts under comparable clinical conditions, without implying individual-level changes.

The 1980–1989 cohort generally showed adjusted median differences that were smaller in magnitude than those observed for the 1990–1999 cohort, often in the same direction. This pattern suggests graded differences between cohorts, while remaining strictly cross-sectional in nature. Across all adjusted models, the interpretation focused on the direction and magnitude of the median differences, with statistical support serving as confirmation rather than the primary descriptor.

An additional pattern observed across the multivariable models concerns the effect of sex. Male patients consistently presented higher adjusted median values for most linear craniofacial measurements compared with females. Importantly, the cohort-associated contrasts persisted after adjustment for sex, indicating that the observed differences across birth cohorts were not explained by sex composition. Future research may further explore whether these cohort-associated patterns vary when analyses are stratified by sex.

A secondary aspect emerges when skeletal classification is considered. Key linear measurements, including maxillary length (Co–A), mandibular length (Co–Gn), and corpus length (Go–Gn), showed lower central values in more recent birth cohorts than in the reference cohort. Because these contrasts persisted after controlling for skeletal class, they did not reflect changes in diagnostic composition but rather differences within each skeletal pattern. Clinically, patients sharing the same sagittal classification may present different absolute linear dimensions depending on the birth cohort group; therefore, values appearing short or long relative to historical norms may still correspond to typical measurements in contemporary patients.

### 3.4. Sensitivity Analyses Across the Distribution

**Figure 2:**
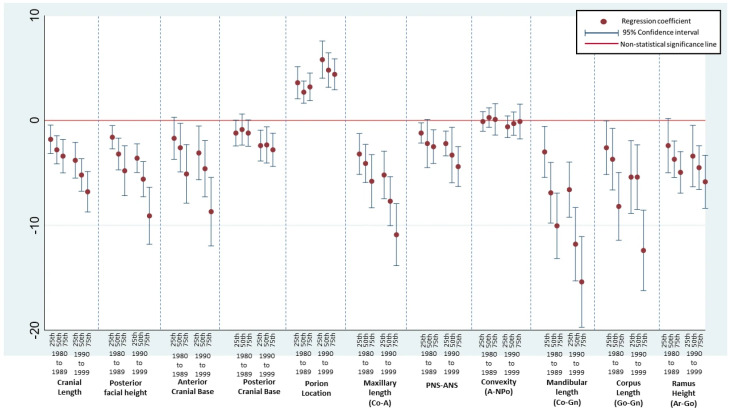
Sensitivity analysis of birth cohort-associated differences across quantiles. Footnote: Adjusted coefficients and 95% confidence intervals from multivariable quantile regression models fitted at the 25th, 50th, and 75th percentiles for the 1980−1989 and 1990−1999 birth cohorts compared with the <1980 reference cohort. The <1980 cohort served as the reference category in the regression models and, therefore, is not displayed in the figure. The vertical axis represents the regression coefficients (mm), scaled from −20 to 10. The horizontal red line indicates coefficients equal to zero. Coefficients with both upper and lower limits of the 95% confidence interval below zero indicated statistically significant lower median values relative to the reference cohort, whereas coefficients with both limits above zero indicated statistically significant higher values. Confidence intervals crossing zero indicate non-statistically significant differences between groups.

To assess whether cohort-associated differences were confined to the center of the measurement distributions or varied across the distributions, additional quantile regression analyses were conducted at the 25th, 50th, and 75th percentiles. The adjusted coefficients and 95% confidence intervals across the quantiles are shown in Figure 2. For several measurements, cohort-associated differences were consistent across quantiles, whereas for others, they were more pronounced at specific parts of the distribution. Quantiles with confidence intervals crossing zero indicate distributional segments in which cohort differences were not supported (Figure 2). The birth cohort group >1980 was used as the reference category in the regression models and therefore is not displayed in the figure to facilitate the interpretation of relative differences.

### 3.5. Description of Measurements Relative to Clinical Norms and Birth Cohort Groups

**Figure 3:**
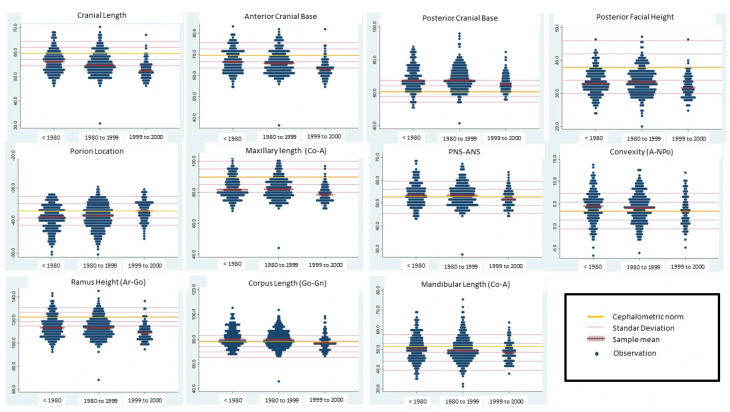
Distribution of selected linear cephalometric measurements across birth cohort groups.  Footnote: Dot plots showing individual linear cephalometric measurements stratified by birth cohort. The central horizontal line represents the reference mean (clinical norm), whereas the additional lines indicate ±1 and ±2 clinical standard deviations, respectively. These graphical representations are descriptive and intended to contextualize the cohort-associated differences within the conventional clinical reference ranges.

Finally, selected linear cephalometric measurements were visualized relative to a fixed clinical reference framework defined by the mean value and ±1 and ±2 standard deviation (SD) thresholds. These graphical displays are descriptive and do not redefine norms. As shown in Figure 3, measurement distributions from more recent birth cohorts were positioned such that values typical of those cohorts more frequently fell below or above the conventional SD-based thresholds derived from earlier cohorts. This visualization illustrates a potential source of under- or overestimation in clinical interpretation when a single reference distribution is applied across patients from different birth cohorts (Figure 3).

## 4. Discussion

This study found that several commonly used linear cephalometric measurements showed distinct central values across adult birth cohort groups, even after adjusting for relevant covariates. These contrasts are cross-sectional and should not be interpreted as individual changes over time. Similar between-group contrasts have been reported in craniofacial and orthodontic research under the concept of secular variation, although many studies do not clearly separate the generational context from individual developmental processes [6,7,9]. By framing the findings as birth cohort-associated distributions within a cross-sectional design, the present analysis aligns with the limitations of clinical records and avoids developmental or evolutionary interpretations [5].

Consistent with the descriptive analyses, several linear dimensions differed across birth cohort groups, while others showed substantial overlap. This pattern indicates distributional differences rather than uniform shifts across the entire cephalometric system. Prior studies have also reported heterogeneous behavior across craniofacial structures, with some variables showing intergenerational differences and others remaining relatively stable, depending on the anatomical region and measurement type [10,14]. These findings support the interpretation that cohort-associated contrasts are measurement-specific rather than generalized.

When examined through multivariable models, several contrasts persisted after adjustment for sex, age group, and sagittal skeletal classification. This is important because previous investigations comparing craniofacial dimensions across populations or generations have not consistently accounted for these clinical modifiers, which can confound interpretation [9,15,16]. The persistence of adjusted distributional contrasts supports an association between birth cohort context and the distribution of certain linear measurements under comparable clinical conditions.

Importantly, cohort-associated differences were not observed in any of the measurements. This heterogeneity is consistent with previous reports, indicating that some cephalometric variables are more sensitive to population context, whereas others demonstrate relative stability across age groups or generations [17]. Studies on monozygotic twins have also reported minimal within-pair variation, supporting high heritability for several linear craniofacial measurements and suggesting that these dimensions do not always vary across contexts [18]. The coexistence of stable and variable measures reinforces the interpretation that the observed contrasts are construct-specific rather than artifactual or indicative of a generalized craniofacial shift.

From a clinical perspective, these differences are relevant when linear measurements are interpreted using fixed reference means and standard deviations. Several authors have emphasized that norms derived from historical samples may not adequately reflect contemporary clinical populations, particularly when cohort-associated variations exist [4,5,6,7,8,19,20]. Visualization relative to conventional ±1 and ±2 standard deviation thresholds shows that values typical of more recent birth cohort groups can occupy different positions within the reference frameworks derived from earlier populations [1,2,21]. This does not indicate an abnormality within the cohort group. Instead, it points to a potential source of systematic over- or underestimation when the birth cohort context is not considered.

These distributional contrasts may reflect generational differences, changes in the characteristics of patients seeking orthodontic care, or broader access to treatment. Each interpretation remains plausible within the context of a cross-sectional design. In addition, longitudinal untreated records are scarce because of ethical reasons. It is not acceptable to diagnose patients who require and request treatment and intentionally leave them untreated to obtain subsequent untreated observations. This constraint increases the practical value of birth cohort group comparisons in routine clinical datasets [12,13] and helps explain why historical untreated growth collections are limited in size and are not representative of current clinical patients [15].

Millimeter-level contrasts in the maxillary and mandibular linear measurements also warrant attention. Even small discrepancies can influence diagnostic classification and perceived skeletal relationships when values are near common thresholds [2,4,22]. Many reports describe intergenerational differences without explicitly addressing how reference-based interpretations shift across birth cohort groups. The present findings support a narrower conclusion. The clinical relevance lies in refining the interpretive context, not in redefining normality.

The study population comprised individuals seeking orthodontic care at a single clinic; records were randomly selected from the available clinical database to reduce selection bias in the treated population. However, the sample was not representative of the Mexican population or Mexico City. This limits the generalization of the findings to the clinical setting, which mirrors real-world applications and supports the practical relevance of the findings [22,23]. Ethnic background was not recorded, and genetic variability was not assessed. However, genomic evidence in Mexico [24] indicates that major genetic differentiation is structured mainly at broad regional scales, whereas large metropolitan populations are highly admixed. In this context, substantial ethnic stratification within an urban clinical center is not expected to align systematically with birth cohort groups. Therefore, unmeasured ethnic variation tends to increase dispersion rather than explain consistent cohort-associated contrasts.

Regarding surgical cases, records were excluded only when previous craniofacial or orthognathic surgery had been performed. Patients with surgical indications at diagnosis were retained to preserve the clinical spectrum encountered in practice. Excluding these patients would restrict variability and reduce internal validity. Adjustment for sagittal skeletal classification further limits the concern that differences reflect changes in case mix rather than cohort-associated distributions.

This study has several limitations. The cross-sectional design supports the evaluation of associations between birth cohort groups and measurement distributions, but it does not support causal inference or individual trajectories. Retrospective clinical records introduce potential selection bias, although this context reflects routine practice and supports its applicability [23,25]. Socioeconomic information was unavailable; therefore, potential access-related or environmental modifiers could not be evaluated directly. Finally, linear measurements were analyzed as predefined independent clinical constructs, and the integrated craniofacial morphology was not assessed.

## 5. Conclusions

This study showed that several routinely used linear cephalometric measurements present different central values across adult birth cohort groups under comparable clinical conditions. These differences persisted after adjusting for clinical covariates and reflected variations in the relative position of measurements within reference distributions rather than individual growth or temporal change. Clinically, fixed reference means and standard deviations may therefore lead to systematic under- or overestimation of linear dimensions in adults from different birth cohorts, supporting a cohort-aware interpretation of routine cephalometric assessment.

## Figures and Tables

**Table 1:** Definitions of linear cephalometric measurements.

Measure	Definition	Normal Value	Standard Deviation
Anterior Cranial Base (SN)	Distance in millimeters from the Center of cranium (CC) point to the Nasion point (NA)	71 mm	±3 mm
Posterior Cranial Base (S–Ar)	Distance from Sella (S) to Articulare (Ar), representing posterior cranial base length	34 mm	±3 mm
Posterior Facial Height (Go–CF)	Distance in millimeters from the middle of the inferior contour of the angle of the mandible located by the ramus (Go) and projected towards the chin to the point of intersection of the pterygoid vertical to the Frankfurt horizontal plane (CF)	62 mm	±4 mm
Porion Location	Distance in millimeters from the Pterygoid vertical (PTV) to the porion point (Po), where the porion location value is negative, indicating that the porion is located posterior to the PTV	—	—
Maxillary Length (Co–A)	Linear distance from the Condylion (Co) to Point A, indicating the maxillary anteroposterior length	95 mm	±4 mm
PNS–ANS	Distance from the Posterior Nasal Spine (PNS) to the Anterior Nasal Spine (ANS), representing the maxillary size	52 mm	±3 mm
Convexity (A–NPo)	Distance in millimeters from Point A to the Facial plane (NPo), when measured perpendicular to the maxilla	2 mm	±2 mm
Mandibular Length (Co–Gn)	Distance from the Condylion (Co) to the Gnathion (Gn), representing total mandibular length	125 mm	±5 mm
Corpus Length (Go–Gn)	Distance from the Gonion (Go) to the Gnathion (Gn), reflecting the length of the mandibular body	78 mm	±4 mm
Ramus Height (Ar–Go)	Vertical distance from the Articulare (Ar) to the Gonion (Go), indicating the mandibular ramus height	55 mm	±4 mm

**Table 2:** Linear cephalometric measurements according to birth cohort group.

**Variables**	**Birth Cohort Group**				
	**Total**	**<1980**	**1980 to 1989**	**1990 to 1999**	*p*-Value
	**n = 604**	n = 220	**n = 289**	**n = 95**	
**Cephalometric Variables**	**Median (IQR)**	**Median (IQR)**	**Median (IQR)**	**Median (IQR)**	
Cranial Length (mm)	54.6 (51.9; 58.3)	56.2 (52.7; 59.1)	54.6 (52.0; 58.4)	52.4 (50.3; 54.9)	<0.001
Anterior Cranial Base (SN) (mm)	65.2 (61.8; 69.7)	66.4 (62.15; 71.1)	65.7 (61.8; 69.6)	63.0 (60.5; 65.1)	<0.001
Posterior Facial Height (Go-CF) (mm)	67.35 (63.2; 72.9)	67.5 (63.6; 74.0)	67.6 (63.5; 73.4)	65.3 (62.3; 70.3)	0.011
Posterior Cranial Base(S-Ar) (mm)	33.0 (30.6; 35.8)	33.0 (31; 36.1)	33.4 (30.6; 36.1)	31.7 (29.2; 34.5)	0.002
Porion Location (mm)	−38.4 (−41.1; −35.5)	−38.9 (−41.8; −36.2)	−38.5 (−41.2; −35.6)	−37 (−39.3; −34.6)	<0.001
Maxillary Length (Co-A) (mm)	80.9 (77.1; 86.3)	81.7 (78.0; 87.9)	82.0 (77.5; 87.0)	78.5 (75; 82.6)	<0.001
PNS-ANS (mm)	53.1 (49.8; 56.4)	53.4 (50; 56.8)	53.4 (49.9; 56.7)	51.7 (49.1; 54.6)	0.014
Convexity (A-NPo) (mm)	4.1 (1.7; 6.35)	4.5 (2.05; 6.65)	4.1 (1.7; 6.3)	3.3 (0.8; 5.7)	0.055
Mandibular Length (Co-Gn) (mm)	112.25 (106.15; 118.9)	113.3 (106.75; 119.8)	113.2 (107.1; 119.5)	108.9 (103.4; 113.8)	<0.001
Corpus Length (Go-Gn) (mm)	79.1 (75.4; 84.9)	79.8 (76.0; 86.3)	79.6 (75.5; 85.0)	77 (73.5; 80.4)	<0.001
Ramus Height (Ar-Go) (mm)	49.4 (45.6; 53.7)	50.2 (45.5; 54.9)	49.3 (46.0; 53.5)	48.4 (44.6; 52)	0.059

IQR  = Interquartile range; *p*-values correspond to the Kruskal–Wallis test.

**Table 3:** Results of the multivariate quantile regression model at the 50th percentile.

	**Model 1**	**Model 2**	**Model 3**	**Model 4**	**Model 5**	**Model 6**	**Model 7**	**Model 8**	**Model 9**	**Model 10**	**Model 11**
**Variable**	Cranial **Length**	**Anterior Cranial Base (SN)**	**Posterior Facial Height (Go–CF)**	**Posterior Cranial Base (S–Ar)**	**Porion Location**	**Maxillary Length (Co–A)**	**PNS–ANS**	**Convexity (A–Npo)**	**Mandibular Length (Co–Gn)**	**Corpus Length (Go–Gn)**	**Ramus Height (Ar–Go)**
	Birth cohort group										
1900–1979	Ref.	Ref.	Ref.	Ref.	Ref.	Ref.	Ref.	Ref.	Ref.	Ref.	Ref.
1980–1989	−2.8	−3.2	−2.6	−0.87	2.7	−4.1	−2.2	0.28	−6.9	−3.7	−3.7
	(−4.16 to −1.44) ***	(−4.75 to −1.65) ***	(−4.63 to −0.57) *	(−2.20 to 0.47)	(1.64 to 3.76) ***	(−5.88 to −2.32) ***	(−4.34 to −0.06) *	(−0.70 to 1.25)	(−9.86 to −3.94) ***	(−6.49 to −0.91) **	(−5.42 to −1.98) ***
1990–1999	−5.2	−5.6	−4.6	−2.33	4.8	−7.7	−3.3	−0.3	−11.8	−5.4	−4.5
	(−6.75 to −3.65) ***	(−7.21 to −3.99) ***	(−7.08 to −2.12) ***	(−3.93 to −0.74) **	(3.20 to 6.40) ***	(−10.21 to −5.19) ***	(−5.72 to −0.88) **	(−1.47 to 0.87)	(−15.19 to −8.41) ***	(−8.40 to −2.40) ***	(−6.56 to −2.44) ***
	Age group										
18–29	Ref.	Ref.	Ref.	Ref.	Ref.	Ref.	Ref.	Ref.	Ref.	Ref.	Ref.
30–39	−1.6	−2.7	−2.1	−0.67	2.5	−3.9	−1.6	0.55	−5.5	−3	−1.7
	(−2.73 to −0.47) **	(−4.06 to −1.34) ***	(−4.12 to −0.08) *	(−2.12 to 0.79)	(1.48 to 3.52) ***	(−5.88 to −1.92) ***	(−3.30 to 0.10)	(−0.39 to 1.49)	(−7.95 to −3.05) ***	(−5.07 to −0.93) **	(−3.05 to −0.35) *
40–49	−2.6	−2.8	−3.9	−1.77	3.2	−6.5	−3.3	0.7	−10.9	−5.5	−6.2
	(−4.45 to −0.75) **	(−5.89 to 0.29)	(−6.58 to −1.22) **	(−3.34 to −0.19) *	(1.51 to 4.89) ***	(−9.18 to −3.82) ***	(−6.13 to −0.47) *	(−0.70 to 2.10)	(−14.46 to −7.34) ***	(−8.88 to −2.12) **	(−8.60 to −3.80) ***
50–99	−2.3	−4	−3.3	−1.53	2.4	−5.2	−1.9	−0.3	−6.2	−3.5	−2.3
	(−5.39 to 0.79)	(−9.44 to 1.44)	(−8.26 to 1.66)	(−3.52 to 0.45)	(−0.85 to 5.65)	(−9.17 to −1.23) *	(−5.34 to 1.54)	(−1.97 to 1.37)	(−12.24 to −0.16) *	(−8.20 to 1.20)	(−5.52 to 0.92)
	Sex										
Female	Ref.	Ref.	Ref.	Ref.	Ref.	Ref.	Ref.	Ref.	Ref.	Ref.	Ref.
Male	3.8	4.7	7.3	3.7	−2.4	6.4	4	−0.1	8.7	4.6	5
	(2.74 to 4.86) ***	(3.45 to 5.95) ***	(5.76 to 8.84) ***	(2.99 to 4.54) ***	(−3.14 to −1.66) ***	(4.58 to 8.22) ***	(2.87 to 5.13) ***	(−0.73 to 0.48)	(6.72 to 10.68) ***	(2.76 to 6.44) ***	(3.68 to 6.32) ***
	Skeletal class										
Class I	Ref.	Ref.	Ref.	Ref.	Ref.	Ref.	Ref.	Ref.	Ref.	Ref.	Ref.
Class II	−0.2	0.9	0.1	−0.1	−0.6	1.9	2.5	4.8	−2.3	0.10	−1.7
	(−1.23 to 0.83)	(−0.20 to 2.00)	(−1.56 to 1.76)	(−1.13 to 1.00)	(−1.49 to 0.29)	(0.28 to 3.52) *	(1.15 to 3.85) ***	(4.31 to 5.39) ***	(−4.40 to −0.20) *	(−1.30 to 1.50)	(−2.84 to −0.56) **
Class III	−0.5	0.1	−1.7	−1.1	−0.6	−2.8	−0.2	−2.5	0.9	3.2	−0.6
	(−3.05 to 2.05)	(−2.03 to 2.03)	(−6.12 to 2.72)	(−4.45 to 2.31)	(−2.58 to 1.38)	(−5.40 to −0.20) *	(−2.24 to 1.84)	(−3.39 to −1.66) ***	(−4.42 to 6.22)	(−2.05 to 8.45)	(−3.80 to 2.60)
Constant	53	62.3	61.4	29.67	−37.8	76.6	48	−0.03	110.8	77.4	48.4
	(51.17 to 54.83) ***	(60.21 to 64.39) ***	(58.14 to 64.66) ***	(27.78 to 31.55) ***	(−39.55 to −36.05) ***	(73.26 to 79.94) ***	(45.08 to 50.92) ***	(−1.23 to 1.18)	(106.67 to 114.93) ***	(74.12 to 80.68) ***	(45.78 to 51.02) ***

Coef, coefficient; 95% CI, 95% confidence interval; Ref, Reference category; NA, not applicable * *p* = 0.05, ** *p* = 0.01, *** *p* = 0.001.

## Data Availability

The raw data supporting the conclusions of this article will be made available by the authors on request.

## Abbreviations

The following abbreviations are used in this manuscript:

S	Sella
N	Nasion
PNS	Posterior Nasal Spine
ANS	Anterior Nasal Spine
Go	Gonion
CF	Frankfurt plane
Gn	Gnathion
A	Point A
NPo	Nasion pogonion line
PL	Porion Location
ACL	Anterior Cranial Length
FH	Anterior Cranial Base (SN)
CF	Cranial Floor
PPL	Palatal Plane (PNS–ANS)
PFH	Posterior Facial Height (Go–CF)
MCL	Mandibular Corpus Length (Go–Gn)
A–NPo	Convexity (A–NPo)
S–Ar	Posterior Cranial Base
Co–A	Maxillary Length
Co–Gn	Mandibular Length
Ar–Go	Ramus Height
